# Exploring the diversity-stability paradigm using sponge microbial communities

**DOI:** 10.1038/s41598-018-26641-9

**Published:** 2018-05-30

**Authors:** Bettina Glasl, Caitlin E. Smith, David G. Bourne, Nicole S. Webster

**Affiliations:** 1 Australian Institute of Marine Science, Townsville, Qld Australia; 2 College of Science and Engineering, James Cook University, Townsville, Qld Australia; 3 AIMS@JCU, Townsville, Qld Australia; 4 Australian Centre for Ecogenomics, University of Queensland, Brisbane, Qld Australia

## Abstract

A key concept in theoretical ecology is the positive correlation between biodiversity and ecosystem stability. When applying this diversity-stability concept to host-associated microbiomes, the following questions emerge: (1) Does microbial diversity influence the stability of microbiomes upon environmental fluctuations? (2) Do hosts that harbor high *versus* low microbial diversity differ in their stress response? To test the diversity-stability concept in host-associated microbiomes, we exposed six marine sponge species with varying levels of microbial diversity to non-lethal salinity disturbances and followed their microbial composition over time using 16S rRNA gene amplicon sequencing. No signs of sponge stress were evident following salinity amendment and microbiomes exhibited compositional resistance irrespective of their microbial diversity. Compositional stability of the sponge microbiome manifests itself at distinct host taxonomic and host microbial diversity groups, with (1) stable host genotype-specific microbiomes at oligotype-level; (2) stable host species-specific microbiomes at genus-level; and (3) stable and specific microbiomes at phylum-level for hosts with high *versus* low microbial diversity. The resistance of sponge microbiomes together with the overall stability of sponge holobionts upon salinity fluctuations suggest that the stability-diversity concept does not appear to hold for sponge microbiomes and provides further evidence for the widely recognized environmental tolerance of sponges.

## Introduction

Marine invertebrates establish relationships with a wide diversity of microorganisms that undertake fundamental roles in host nutrition, waste-product removal, host immunity, pathogen defense and host development^1–3^. The ecological unit comprised of the animal host and its associated microbes is often referred to as a holobiont^4,5^, where the associated microbes are not a random aggregation of environmental microorganisms but rather a selected consortium, critical to the well-being of the host^1,6^. Disturbances or changes in the environment can destabilize the microbiome, often with adverse consequences for host health^7–9^.

The application of concepts developed for the field of community ecology can be useful to better understand environmental drivers of microbial community dynamics^10,11^. Similar to ecological communities^12^, microbial communities can respond to disturbance events in different ways^13^. For example, a microbiome can be entirely resistant to a stressor and hence no change in the community composition occurs^14–16^. Alternatively, resilient microbial communities may shift immediately following the disturbance event but return to their original composition once the stressor(s) has been removed^7^. However, if the shift is too dramatic or the original composition cannot be restored, the holobiont homeostasis can collapse which is often associated with disease and/or host mortality^7,15,17,18^. The type of response a microbiome will exhibit upon disturbance is difficult to predict and likely depends on the nature of host-microbe association (facultative *versus* obligate), plus the strength and/or duration of the disturbance^19^. Another potential factor may be the diversity (defined as richness and evenness) of a microbiome. Increased biodiversity, for example, has been postulated to increase the stability of an ecosystem^20^. For hosts associated with highly diverse microbiomes, these associations may provide greater functional repertoires and functional redundancies compared to animals that host less diverse microbiomes.

The association between sponges and their microorganisms represents one of the most evolutionarily ancient examples of symbiosis in multicellular life^2,21^. The diversity of microorganisms within sponges varies considerably amongst species^22,23^ and between sponges that host high (high microbial abundance; HMA) or low (low microbial abundance; LMA) microbial abundance^24,25^. In general, microbial composition also differs between HMA and LMA species, with LMA sponges being dominated by Proteobacteria and Cyanobacteria^26–28^ and HMA sponges being dominated by the phyla Chloroflexi, Acidobacteria, Actinobacteria and PAUC34f^25^. HMA and LMA sponge species are also thought to differ in their functional gene content^29^, pumping rates^30^, and their cycling of carbon and nitrogen compounds^31^. Although notable similarities in microbiome stability over seasonal scales has been detected across the HMA-LMA dichotomy^28^, how microbial diversity and abundance affects sponge microbiome stability upon acute environmental fluctuations has not yet been defined.

**Figure 1:**
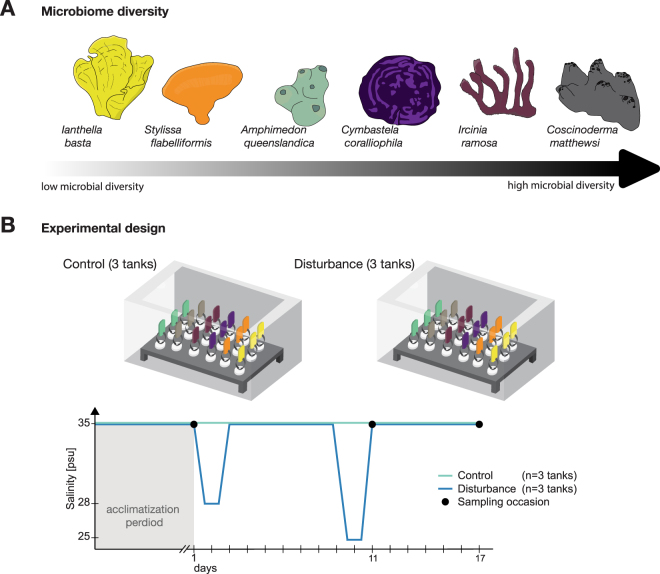
Diversity of sponge microbiomes and experimental setup to test microbiome stability. (**A**) Sponge microbiomes vary substantially in their diversity, ranging from very low (Shannon index of approximately 1.3) to very high (Shannon index of approximately 4.9) microbial diversity. (**B**) In total, six sponge genotypes per species were collected and each genotype was fragmented into three equally sized clones. Clones of each genotype were placed into the same experimental tanks to enable sub-sampling over time. The experimental design comprised three control tanks and three disturbance tanks, with each tank containing 18 sponge clones in total. Sponge clones were acclimatized to experimental conditions for one week and then one clone/genotype was sampled across all tanks immediately prior to the first disturbance event. One additional clone/genotype was sampled for each experimental tank 24 h and 168 h after the second pulse disturbance. Sponges in disturbance tanks experienced two consecutive salinity drops (28 psu and 25 psu, respectively), whereas sponges in control tanks were maintained at stable ambient salinity (35 psu) over the duration of the experiment.

This study investigates how the diversity of the sponge microbiome influences community stability upon acute salinity fluctuations (ranging from 36 psu to 25 psu) under controlled experimental conditions (Fig. 1). The simulated fluctuation mimics natural salinity levels experienced by reef organisms after major flooding events^32,33^, and therefore provides further insights into the environmental tolerance (ability to live within a certain range of abiotic factors) of sponge holobionts to short-term salinity stress. Stability was investigated for six marine sponge species (*Amphimedon queenslandica*, *Ianthella basta* and *Stylissa flabelliformis* as representatives of low microbial diversity species; and *Coscinoderma matthewsi, Cymbastela coralliophila* and *Ircinia ramosa* as representatives of high microbial diversity species) using high taxonomic resolution based on Amplicon Sequence Variants (ASV)^34^, facilitating detection of fine-scale variations in microbiome composition.

## Results

### Host health and photopigment composition

Sponges were not visibly stressed following salinity amendment as determined using the previously described stress proxies of mucus production, tissue regression and tissue necrosis^15^. Photopigment concentrations (Chlorophyll *a*, *b*, *c*, *d*, total chlorophyll and total carotenoids) were evaluated for each species as an additional proxy of host health (Supplementary Material, Figure S1). Photopigment concentrations varied significantly between host species (ANOVA, F_(5/630)_ = 8.145, p = 1.84^−7^). *S. flabelliformis* had the highest total carotenoid concentration (150.57 µg g^−1^ ± 48.51) followed by *I. basta* (41.41 µg g^−1^ ± 9.48). Chlorophyll *a* concentration was highest in the two photosynthetic species *I. ramosa* and *C. coralliophila*, ranging from 100.63 µg g^−1^ ± 37.60 to 97.20 µg g^−1^ ± 33.79 respectively. Neither time nor treatment had an effect on the photopigment composition within each host species (PERMANOVA, p > 0.05, Table S1).

### Microbiome diversity and richness

**Figure 2:**
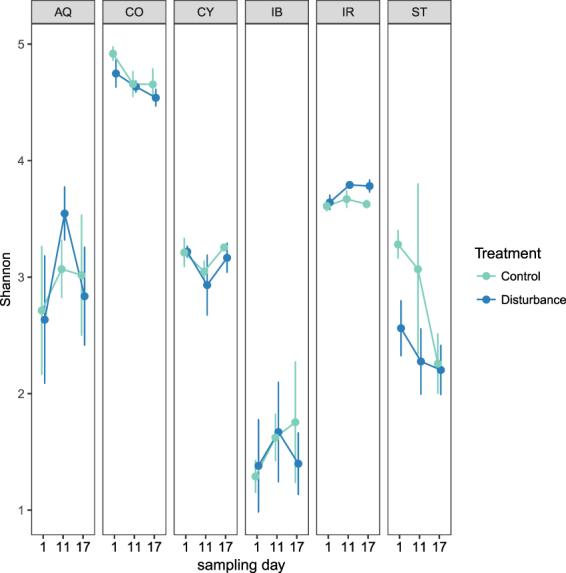
Variation in Shannon diversity (mean ± SD) in each sponge species across treatments and sampling times. *Amphimedon queenslandica* (AQ), *Coscinoderma matthewsi* (CO), *Cymbastela coralliophila* (CY), *Ianthella basta* (IB), *Ircinia ramosa* (IR) and *Stylissa flabelliformis* (ST).

In total, 7 077 372 Illumina sequence reads were obtained (ranging from 5 976 to 57 917 in the different samples), of which 3 185 811 reads remained after quality filtering. Overall, 6 896 ASV were identified based on single nucleotide variations in the sequence reads. The highest richness was observed in *A. queenslandica* (297 ASVs ± 94), while *I. basta* was associated with the lowest microbial richness (66 ASVs ± 62) (Table S2). Alpha diversities based on Shannon Index varied significantly between sponge species (ANOVA, F_(5/72)_ = 85.356, p = 2 × 10^−16^, Table S3; Fig. 2). *C. matthewsi* was associated with the highest alpha diversity (4.69 ± 0.18), followed by *I. ramosa* (3.69 ± 0.10), *C. coralliophila* (3.14 ± 0.23), *A. queenslandica* (2.97 ± 0.71) and *S. flabelliformis* (2.61 ± 0.68). *I. basta* associated microbiomes had the lowest microbial diversity (1.52 ± 0.54). Sponges from the different treatment groups (control *versus* disturbance) had similar diversity values, indicating acute salinity disturbance had no influence on microbiome richness or evenness within each sponge species (Fig. 2).

### Compositional stability of sponge microbiomes after salinity fluctuations

**Figure 3:**
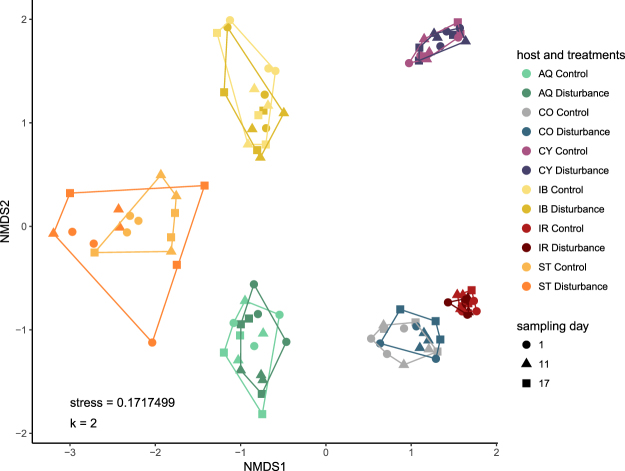
Non-metric multidimensional scaling plot displaying similarities in the microbiomes of the six sponge species under both treatment conditions (control and disturbance). Microbiomes show high host-species specificity and high temporal stability even after exposure to a non-lethal salinity stress. Abbreviation of the host species as indicated: *Amphimedon queenslandica* (AQ), *Coscinoderma matthewsi* (CO), *Cymbastela coralliophila* (CY), *Ianthella basta* (IB), *Ircinia ramosa* (IR) and *Stylissa flabelliformis* (ST).

**Figure 4:**
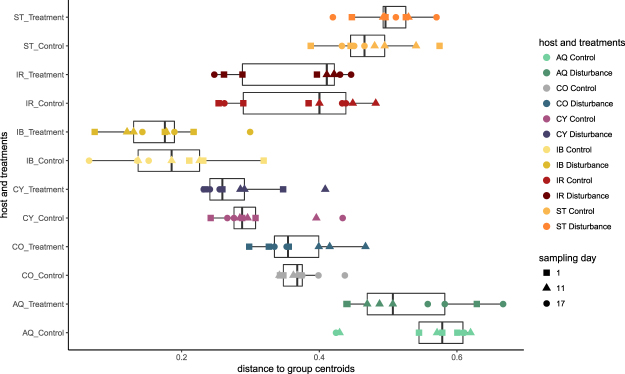
Microbiome variability (heterogeneity) for *Amphimedon queenslandica* (AQ), *Coscinoderma matthewsi* (CO), *Cymbastela coralliophila* (CY), *Ianthella basta* (IB), *Ircinia ramosa* (IR) and *Stylissa flabelliformis* (ST) under both treatment conditions (control and disturbance) including all sampling points (day 1, day 11 and day 17). Distance to group centroid (also referred to as dispersion), is used to describe heterogeneity in the microbiome.

The stability of the sponge microbiome upon two consecutive pulses of reduced salinity was compared across HMA and LMA species. Each sponge species was associated with a distinct microbial community (ANOSIM, p = 0.001, R = 0.9793) and microbiomes of both treatment groups (control *versus* disturbance) were highly similar within each sponge species (ANOSIM p = 0.027, R = −0.0070; Fig. 3). Multivariate dispersion (heterogeneity of a community based on distances of samples to their group centroid) of microbial assemblages varied significantly between sponge species (ANOVA, F_(11/96)_ = 42.383, p = 2.2 × 10^−16^; Fig. 4), however, treatment had no effect on the dispersion of the sponge microbiome (TukeyHSD p > 0.05, Table S4). Microbial community composition in each sponge species also remained stable over time within each treatment group (adonis2, host and treatment group as blocking factor, 10 000 permutations, p = 0.9989, Table S5). However, host genotype had a significant effect on microbial composition for all sponge species, with a higher similarity between samples originating from the same genotype than between samples originating from different conspecific genotypes (ANOSIM, p = 0.001, R = 0.9427). Furthermore, the microbiome composition varied significantly between sponge individuals (genotypes) of the same species (adonis2, host species as blocking factor, 10 000 permutations, p < 0.001, Table S6).

### Fine-scale variations in sponge microbiomes

**Figure 5:**
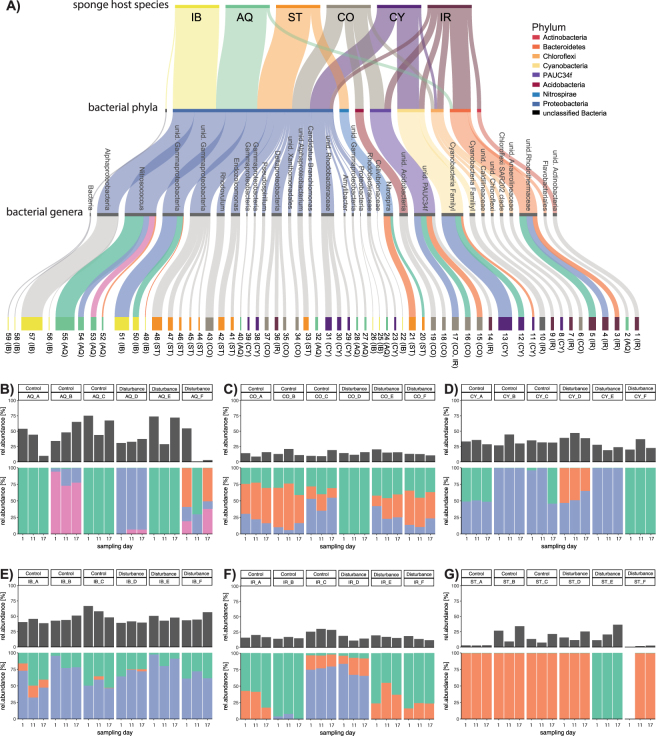
(**A**) Alluvial diagram depicting taxonomic affiliation of the ten most abundant Amplicon Sequence Variants (ASV) associated with each sponge species (AQ = *Amphimedon queenslandica*, CO = *Coscinoderma matthewsi*, CY = *Cymbastela coralliophila*, IB = *Ianthella basta*, IR = *Ircinia ramosa* and ST = *Stylissa flabelliformis*). Colour of ASV nodes represent host species (AQ = green, CO = grey, CY = purple, IB = yellow, IR = red, ST = orange). (**B**–**G**) Fine-scale compositional variation of selected bacterial taxa associated with host genotypes. (**B**) *Nitrosococcus* ASV associated with AQ genotypes. (**C**) PAUF34f ASV associated with CO genotypes. (**D**) Cyanobacteria Family I ASV associated with CY genotypes. (**E**) unid. Gammaproteobacteria associated with IB genotypes. (**F**) *Rhodothermaceae* ASV associated with CY genotypes and (**G**) *Nitrospira* ASV associated with ST genotypes.

Sponge microbiomes were dominated by sequences classified to the phyla Proteobacteria, Chloroflexi, Cyanobacteria, Bacteroidetes and PAUC34f (Fig. 5A). The ten most abundant ASVs for each sponge species are represented in Fig. 5A and the ASV composition for selected taxa is shown for each host genotype in Fig. 5B. *A. queenslandica* was dominated by seven genera belonging to the phyla Proteobacteria and Bacteroidetes (Fig. 5A). *Nitrosococcus* (phylum Proteobacteria) was the most abundant genus and was represented by four ASVs (Fig. 5B). Each *A. queenslandica* host genotype was associated with a specific *Nitrosococcus* community (ANOSIM, p = 0.001., R = 0.7128), which displayed high temporal stability irrespective of treatment. *C. matthewsi* was dominated by six genera belonging to Proteobacteria, PAUC34f, Chloroflexi and Acidobacteria phyla (Fig. 5A). The three most abundant PAUC34f ASVs retrieved from the *C. matthewsi* microbiome were equally abundant in all host genotypes, except genotype CO_D which was dominated by a single PAUC34f ASV (Fig. 5C). *C. coralliophila* was dominated by seven genera belonging to the phyla Proteobacteria, Cyanobacteria and Chloroflexi with the cyanobacterial ASVs revealing high host genotype specificity and high temporal stability irrespective of treatment (Fig. 5A,D). *I. basta* was dominated by one Alphaproteobacteria-affiliated sequence across all genotypes while the other dominant class, Gammaproteobacteria, consisted of two equally abundant ASVs and a third low abundant ASV which was not present across all host genotypes (Fig. 5A,E). *I. ramosa* was dominated by seven bacterial genera belonging to six phyla, with the most abundant members belonging to *Rhodothermaceae* (phylum Bacteroidetes) (Fig. 5A). *Rhodothermaceae* ASVs varied significantly between the *I. ramosa* host genotypes but were stable within each genotype (Fig. 5F). The *S. flabelliformis* microbiome was dominated by the phyla Proteobacteria and Nitrospirae (Fig. 5A), with the two dominant *Nitrospira* ASVs displaying similar relative abundance patterns across all genotypes except ST_E (Fig. 5G).

## Discussion

Disturbance of the global climate system as a result of increased green-house gas emissions is predicted to result in stronger storm activity and larger flooding events^35^. For near-shore coral reefs, large floods can result in acute salinity fluctuations that impact the health of marine invertebrates such as corals and sponges^32,36^. For example, a flood plume associated with tropical cyclone “Tash” in 2011 caused a dramatic salinity drop (reaching extremes of 6.5 psu) on coral reefs in Keppel Bay (GBR, Australia) which resulted in large-scale coral mortality^36^. Similar salinity extremes and mortalities were observed after cyclone “Joy” crossed the Queensland (Australia) coast in 1991, where salinity during the flood peak reached 7–10 psu at the surface and 15–28 psu at 3 m depth^32^. However, despite experiencing an average annual salinity of ~35.7 psu in the field^37^, sponge species assessed in this study were highly tolerant of short-term acute salinity fluctuations (minimum of 25 psu), showing no visual signs of health deterioration, no changes in the concentration or composition of photopigments and no shifts in the sponge-associated microbial communities. The only previous assessment of salinity tolerance in sponges showed that *Cymbastela concentrica* tolerated long-term exposure to salinities ranging from 30.6 psu to 34.5 psu^38^. These results contribute to an increasing body of evidence showing high environmental tolerance in sponges^39,40^.

The diversity-stability hypothesis posits that high diversity systems are more stable than low diversity systems upon environmental fluctuation^20^. Applying this diversity-stability paradigm to sponge microbiomes subjected to acute salinity disturbance revealed no shift in the compositional stability (e.g. compositional resistance, resilience and sensitivity differences) of the microbiome for both high (HMA) and low (LMA) diversity species. Temporal stability in HMA- and LMA-sponge microbiomes has been described along natural seasonal fluctuations^28^ and sponge microbiomes have also been shown to be resistant to sub-lethal increases in nitrogen, temperature, sediment, light and pollution^14,15,41–44^. Furthermore, sponge microbiomes can remain stable during stress-induced tissue regression of the host^45^. However, once a compositional and functional shift of the sponge-associated microbiome occurs, host mortality can rapidly follow^17,18,46^, highlighting the crucial link between microbial stability and host health. In addition to altering the abundance and/or prevalence of microorganisms, environmental disturbances can also induce changes to the community dispersion/heterogeneity^47^. The recently coined Anna Karenina principle postulates that disturbances often lead to more stochastic community structures^47^, which can be measured by the increase in multivariate dispersion of a microbiome. In our study the dispersion of microbial communities also remained consistent across both high and low diversity species, irrespective of experimental treatment. Stability in the composition and dispersion of sponge-microbial associations under short-term salinity stress emphasizes the high fidelity of sponge-microbial partnerships. Furthermore, equal compositional resistance across high and low microbial diversity species during environmental fluctuations shows that the stability of sponge microbiomes remains unaffected by its diversity. While the diversity-stability concept does not appear to hold for sponge microbiomes, it remains to be seen whether the environmental tolerance of other reef species such as corals is linked to microbiome diversity. Furthermore, the effect of microbial diversity on functional stability of sponge microbiomes remains to be determined.

Oligotyping sequence clustering techniques identify nucleotide variations (up to one nucleotide) between sequences and hence increase the ability to detect fine-scale variations, which can be informative about ecological niches, temporal dynamics and population structures^48–50^. In this study, oligotyping revealed that host genotype significantly controls fine-scale bacterial composition (ASV level), whereas sponge species structures the associated bacterial genera, and the HMA-LMA dichotomy appears to influence the microbiome composition at the phylum level (Fig. 5). For example, low microbial diversity species (*A. queenslandica*, *I. basta* and *S. flabelliformis*) were predominantly associated with bacteria belonging to the phylum Proteobacteria. In contrast, high microbial diversity species (*C. matthewsi*, *C. coralliophila* and *I. ramosa*) were associated with a complex community dominated by Proteobacteria, PAUC34f, Chloroflexi, Bacteroidetes, Actinobacteria and Acidobacteria. Similar observations have been reported for other LMA and HMA sponge species^25^ and results are also consistent with previous reports of high species-specificity in sponge microbiomes^28,51–53^. Here we further report that sponge microbiomes also exhibit strong genotype-specificity, detected using fine-scale compositional variation at the ASV level. This is consistent with other host-microbe systems including the human gut^54–56^ and the *Drosophila* microbiome^57,58^. Considering the significant microbiome differences amongst host genotypes, we argue that future research on sponge microbiomes should take genotype-specific microbiome variations into account. The significant influence of host genotype on the fine-scale composition of a sponge microbiome further suggests that host intrinsic factors (e.g. host genetics) rather than environmental factors are particularly important in shaping the sponge microbiome.

Marine ecosystems, such as coral reefs, are increasingly impacted by local and global stressors^59^ and effective monitoring and management are critical to their protection. Microbial diagnostics have recently been proposed as a rapid and sensitive way to monitor environmental fluctuations in coral reef ecosystems^60^. As ecologically important filter feeders with well-established microbial partnerships^39,61,62^, sponges represent a relevant target for microbial based monitoring approaches. However, the high stability of sponge microbiomes towards a variety of natural fluctuations^28,63^ and stressors^42–44^, in conjunction with fine-scale compositional variation between host genotypes, suggests that sponge-associated microbes are not suitable indicators for assessing perturbations to reef ecosystem health. Here we have also shown that the primary driver of the remarkable stability in sponge-associated microbial communities is environmental resistance rather than resilience.

## Methods

### Experimental setup

Great Barrier Reef (GBR) sponge species (n = 6) associated with previously documented low and high diversity microbial communities^53,64^ were selected for the study and included: *Amphimedon queenslandica*, *Ianthella basta* and *Stylissa flabelliformis* as representatives of low microbial diversity species; and *Coscinoderma matthewsi, Cymbastela coralliophila* and *Ircinia ramosa* as representatives of high microbial diversity species. In total, six individuals of each sponge species were collected from Magnetic Island (*C. matthewsi* and *A. queenslandica*, Australia) and Davies Reef (*C. coralliophila*, *I. basta*, *I. ramosa* and *S. flabelliformis*; Australia) in February 2017. Samples were collected under the permits G12/35236.1 and G16/38348.1 granted by the Great Barrier Reef Marine Park Authority to the Australian Institute of Marine Science. All sponges were immediately transferred to the National SeaSimulator at the Australian Institute of Marine Science (Townsville, Australia), where sponges were kept in flow-through outdoor tanks under natural lighting. Within two days of collection, each sponge was fragmented into three equally sized clones and placed into indoor flow-through tanks for two weeks to allow tissue healing. Sponge clones were subsequently transferred to experimental tanks and left to acclimatize for seven days. Each experimental tank harbored six sponge species, each represented by three clones of the same individual (in total 6 × 3 sponge clones per tank; see Fig. 1).

The experimental setup comprised three control tanks and three pulse salinity disturbance tanks. All tanks were kept at stable temperature (27.5 °C ± 0.04 °C), light (80 mol photons m^−2^ s^−1^) and flow (8 m s^−1^) conditions throughout the experiment. While control tanks were kept at stable ambient salinity (34.77 psu ± 1.05 psu), disturbance tanks were exposed to two consecutive pulse salinity drops on the second (day 2) and tenth day (day 10) to 28 psu and 25 psu, respectively (Fig. 1). Each pulse lasted for a total of nine hours with the intensity and duration of the simulated salinity fluctuations based on previously documented salinity fluctuations on the GBR^32,33,65^. Samples were collected before the disturbance (day 1), directly after the second low-salinity pulse event (day 11) and one week after the pulse event to assess recovery (day 17). On each sampling occasion one clone of each individual sponge was removed from the tanks with sterile tweezers, photographed, rinsed with 0.2 µm filtered seawater to remove loosely attached microbes from the surface and cut into small fragments. Randomly selected subsamples containing pinacoderm and mesohyl were placed into two 2 ml cryogenic vials (Corning^®^), snap frozen in liquid nitrogen and stored at −80 °C until further processing.

### Pigment analysis

The concentration of sponge photopigments was analyzed following the method described by Pineda, *et al*.^15^. Briefly, sponge samples were defrosted, wet weight of each sample was recorded (approximately 0.2 g) and samples were transferred into clean PowerBead tubes (MoBio Power Plant Kit) containing four stainless steel beads per vial. To each tube 1 ml of 95% EtOH was added, and tissue was bead beaten for 3 × 40 s at 5 m s^−1^ and centrifuged for 30 s at 10 000 rcf. The supernatant was added in triplicate into 96-well plates and absorbance was measured at 470 nm, 632 nm, 649 nm, 665 nm, 696 nm and 750 nm on a Bio-Tek® Power Wave Microplate Scanning Spectrophotometer. Blank-corrected absorbance readings were used to calculate Chlorophyll a, b, c, d, total Chlorophyll and total Carotenoids (Supplementary Material). Pigment concentration was normalized to sponge wet weight.

### DNA extraction and sequencing

DNA was extracted from all sponge samples using the MoBio Power Soil Kit following the manufacturer’s instructions, including one bead beating step of 40 s at 4 m s^−1^. DNA extracts were stored at −80 °C until shipment on dry ice to Ramaciotti Centre (University of New south Wales, Australia) for sequencing. The V1-V3 region of the 16S rRNA gene was amplified using primers 27 F (Lane 1991) and 519 R (Lane *et al*. 1993) and libraries were prepared with the Illumina TruSeq preparation protocol, followed by Illumina MiSeq2 × 300 bp sequencing.

### Sequence analysis

Demultiplexed paired end reads were analyzed using QIIME2 (Version 2017.9.0; https://qiime2.org). Based on quality plots, forward and reverse reads were truncated at their 3′ end at the 296 and 257 sequencing positions, respectively. Samples were individually checked for chimeras and chimeric sequences were removed from the dataset using DADA2^66^. Sequences were grouped into features based on 100% sequence similarity, subsequently referred to as ASV (amplicon sequence variants), using DADA2^66^. Multiple *de novo* sequence alignments of the representative sequences was performed using MAFFT^67^. Nonconserved and highly gapped columns from the alignment were removed using default settings of the mask option in QIIME2. Unrooted and rooted trees were generated using FastTree for analysis of phylogenetic diversity. For taxonomic assignment, a Naïve-Bayes classifier was trained on the SILVA v123 99% Operational Taxonomic Units, where reference sequences only included the V1-V2 regions (27 F/519 R primer pair) of the 16S rRNA genes. The trained classifier was applied to the representative sequences to assign taxonomy. Chloroplast and Mitochondria derived sequence reads and singletons were removed from the dataset and the feature table was rarefied to an even sequencing depth of 5976 sequencing reads, representing 21.41% of the total sequences post quality control.

Statistical analyses were performed in R^68^. Multivariate statistical approaches including Analysis of Similarity (ANOSIM, ‘vegan package’^69^), Permutation Multivariate Analysis of Variance (PERMANOVA, ‘vegan package’^69^), Multivariate Homogeneity of Group Dispersion/variance (‘vegan package’^69^) and Non-metric Multidimensional Scaling (NMDS, ‘phyloseq package’^70^) were based on Bray Curtis dissimilarities. Graphs were created in R using ggplot2^71^ and phyloseq packages^70^. The alluvial diagram was generated in RAWGraph^72^.

### Data Availability

Demultiplexed sequences and metadata are available from the Sequence Read Archives (SRA) under accession number SRP131926.

## Electronic supplementary material

Supplementary Material
