# Supplementary material for: Exploring the diversity-stability paradigm using sponge microbial communities
Source: Sci Rep. 2018 May 30;8:8425. doi: 10.1038/s41598-018-26641-9 (PMC5976656; doi:10.1038/s41598-018-26641-9)
Supplement: Supplementary file 1 — Supplementary Material [file 41598_2018_26641_MOESM1_ESM.pdf]

Exploring the diversity-stability paradigm using sponge microbial communities

Bettina Glasl, Caitlin E. Smith, David G. Bourne and Nicole S. Webster

Equations for photopigment concentration

Chlorophyll a, b, c and d, and total chlorophyll and total carotenoid concentrations (in µg/mL) were calculated using the following equations (Lichtenthaler 1987, Ritchie 2008):

$$Chl\ a = \frac{[(-0.9394 \text{ x E632}) + (-4.2774 \text{ x E649}) + (13.3914 \text{ x E665})]}{0.794}$$

$$Chl\ b = \frac{[(-4.0937\ x\ E632) + (25.6865\ x\ E649) + (-7.3430\ x\ E665)]}{0.794}$$

$$Chl\ c = \frac{[(28.5073\ x\ E632) + (-9.9940\ x\ E649) + (-1.9749\ x\ E665)]}{0.794}$$

$$Chl\ d = \frac{[(-0.2007\ x\ E632) + (0.0848\ x\ E649) + (-0.1909\ x\ E665) + (12.1302\ x\ E696)]}{0.794}$$

$$Total\ Chl = \frac{[(24.1209\ x\ E632) + (11.2884\ x\ E649) + (3.7620\ x\ E665) + (5.8338 x E696)]}{0.794}$$

$$Total\ carotenoids = \frac{[((1000\ x\ E470)\ /\ 0.794) - (2.13\ x\ Chl\ a) - (97.64\ x\ Chl\ b)]}{209}$$

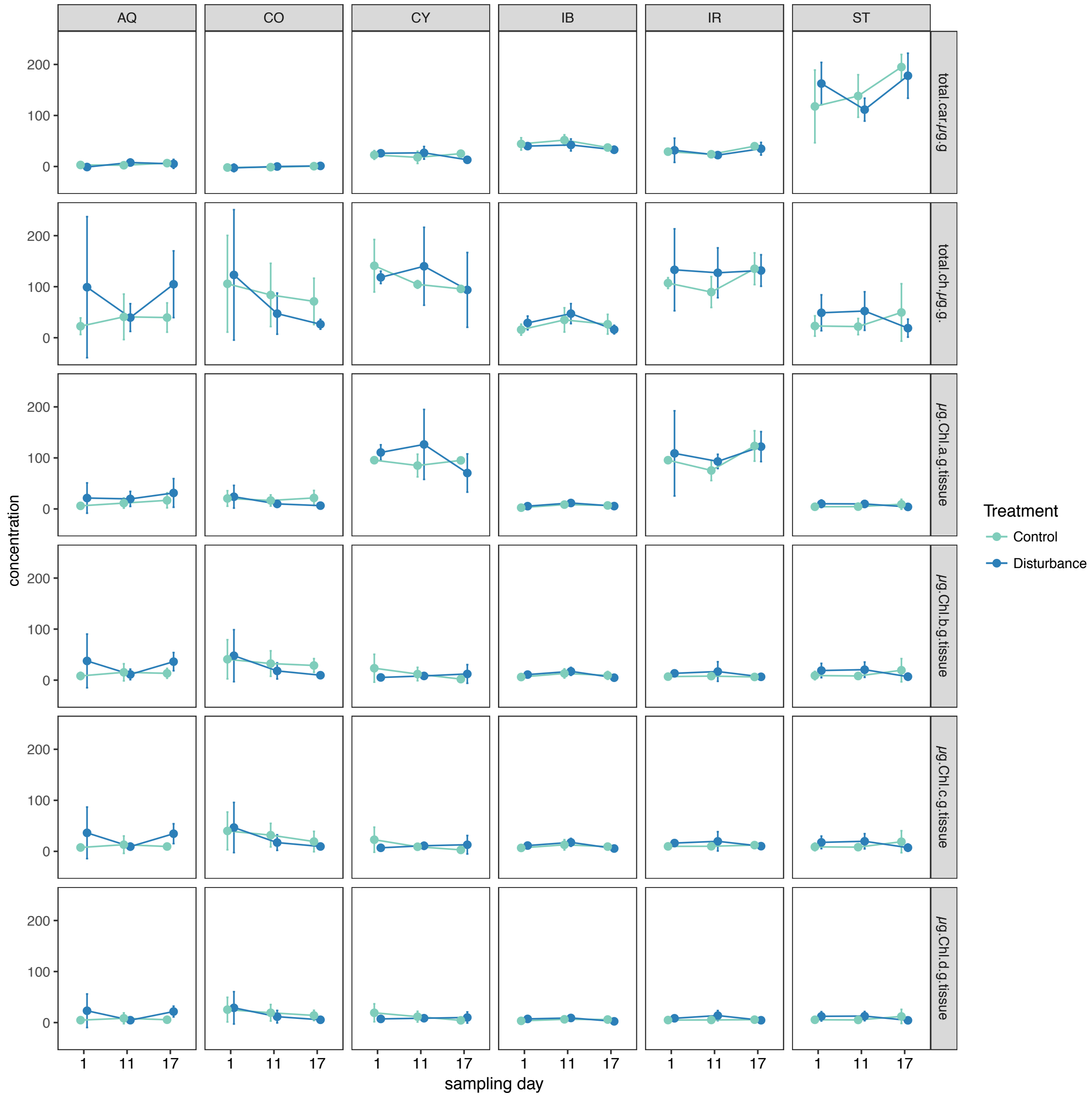

Figure S1: Average photopigment concentration (in µg per g sponge tissue) of control and disturbance samples throughout the experiment. Graph displays the total Carotenoids, total Chlorophyll, Chlorophyll a, b, c and d (from top to bottom) concentration for *Amphimedon queenslandica* (AQ), *Coscinoderma matthewsi* (CO), *Cymbastella coralliophila* (CY), *Ianthella basta* (IB), *Ircinia ramosa* (IR) and *Stylissa flabelliformis* (ST) (from left to right). Error bars represent standard error.

Table S1. Statistical output of PERMANOVA (adonis2, vegan package) testing the effect of treatment and sampling time point on the photopigment composition within each host species (10 000 permutations).

```
adonis2(formula = d ~ Treatment * SamplingTimepoint, data = df, permutations = perm, method = "bray")
```

	Df	SumOfSqs	F	Pr(>F)
Treatment	1	0.0173	0.0987	0.8619
SamplingTimepoint	2	0.0931	0.2657	0.7179
Treatment:SamplingTimepoint	2	0.0650	0.1857	0.8379
Residual	100	17.5122		

Table S2. Overview of microbiome diversity metrics (mean ± standard deviation) for each sponge species for both treatments (control and disturbance) over time. Richness, evenness and Shannon Index were calculated based on a non-rarefied feature table excluding singletons, chloroplast and mitochondria derived reads.

**Host**	**Sampling day**	**Richness**		**Evenness**		**Shannon Index**	
		Control	Disturbance	Control	Disturbance	Control	Disturbance
**AQ**	1	362 (± 137)	257 (± 105)	0.459 (± 0.133)	0.476 (± 0.144)	2.71 (± 0.95)	3.55 (± 0.95)
	11	309 (± 125)	262 (± 68)	0.539 (± 0.051)	0.638 (± 0.045)	3.07 (± 0.42)	3.55 (± 0.39)
	17	307 (± 110)	282 (± 38)	0.532 (± 0.158)	0.505 (± 0.139)	3.02 (± 0.89)	2.84 (± 0.73)
**CO**	1	329 (± 12)	261 (± 53)	0.848 (± 0.012)	0.855 (± 0.010)	4.92 (± 0.10)	4.75 (± 0.20)
	11	263 (± 17)	215 (± 14)	0.836 (± 0.029)	0.863 (± 0.005)	4.66 (± 0.19)	4.64 (± 0.08)
	17	234 (± 55)	201 (± 12)	0.856 (± 0.007)	0.856 (± 0.014)	4.65 (± 0.23)	4.54 (± 0.12)
**CY**	1	99 (± 3)	94 (± 6)	0.699 (± 0.044)	0.709 (± 0.024)	3.21 (± 0.21)	3.22 (± 0.08)
	11	98 (± 7)	82 (± 15)	0.665 (± 0.024)	0.664 (± 0.073)	3.05 (± 0.15)	2.93 (± 0.44)
	17	94 (± 9)	92 (± 11)	0.717 (± 0.012)	0.700 (± 0.031)	3.26 (± 0.02)	3.17 (± 0.22)
**IB**	1	29 (± 3)	51 (± 44)	0.382 (± 0.061)	0.362 (± 0.091)	1.29 (± 0.24)	1.38 (± 0.69)
	11	55 (± 28)	102 (± 99)	0.410 (± 0.043)	0.376 (± 0.081)	1.62 (± 0.35)	1.67 (± 0.74)
	17	87 (± 108)	72 (± 49)	0.438 (± 0.069)	0.334 (± 0.060)	1.75 (± 0.90)	1.40 (± 0.46)
**IR**	1	112 (± 19)	121 (± 10	0.768 (± 0.018)	0.760 (± 0.018)	3.61 (± 0.07)	3.64 (± 0.11)
	11	119 (± 5)	121 (± 11)	0.768 (± 0.031)	0.792 (± 0.009)	3.67 (± 0.12)	3.79 (± 0.02)
	17	110 (± 11)	116 (± 9)	0.772 (± 0.014)	0.795 (± 0.008)	3.63 (± 0.05)	3.78 (± 0.09)
**ST**	1	123 (± 20)	89 (± 11)	0.683(± 0.032)	0.574 (± 0.105)	3.28 (± 0.20)	2.56 (± 0.41)
	11	139 (± 73)	59 (± 19)	0.622 (± 0.188)	0.559 (± 0.085)	3.07 (± 1.27)	2.28 (± 0.49)
	17	95 (± 39)	63 (± 25)	0.504 (± 0.114)	0.538 (± 0.033)	2.26 (± 0.44)	2.20 (± 0.37)

Table S3. Statistical output of ANOVA using to test the differences in alpha diversity (Shannon Index) between treatments, sampling time points and host species.

	Df	Sum Sq	Mean Sq	F value	Pr(>F)	
SamplingTimepoint	2	0.28	0.140	0.589	0.558	
Treatment	1	0.26	0.259	1.090	0.300	
Host	5	101.48	20.297	85.356	<2e-16	***
SamplingTimepoint:Treatment	2	0.04	0.020	0.083	0.921	
SamplingTimepoint:Host	10	3.15	0.315	1.324	0.234	
Treatment:Host	5	1.13	0.226	0.950	0.454	
SamplingTimepoint:Treatment:Host	10	1.06	0.106	0.445	0.919	
Residuals	72	17.12	0.238			

Table S4. Statistical output of the TukeyHSD post hoc test (95% confidence interval) used to compare group dispersion variances between host species and treatment groups. Treatment groups within a host species are highlighted in grey.

Host_Treatment	diff	lwr	upr	p adj
AQ_Treatment-AQ_Control	0.036448345	-0.057054000	0.1299506905	0.9764472
CO_Control-AQ_Control	-0.145971784	-0.239474129	-0.0524694384	0.0000615
CO_Treatment-AQ_Control	-0.163788931	-0.257291276	-0.0702865853	0.0000040
CY_Control-AQ_Control	-0.258198351	-0.351700696	-0.1646960058	0.0000000
CY_Treatment-AQ_Control	-0.196917228	-0.290419573	-0.1034148823	0.0000000
IB_Control-AQ_Control	-0.311864954	-0.405367299	-0.2183626084	0.0000000
IB_Treatment-AQ_Control	-0.382482895	-0.475985241	-0.2889805499	0.0000000
IR_Control-AQ_Control	-0.109206085	-0.202708430	-0.0157037395	0.0088613
IR_Treatment-AQ_Control	-0.193376531	-0.286878876	-0.0998741853	0.0000000
ST_Control-AQ_Control	-0.062353112	-0.155855457	0.0311492338	0.5298100
ST_Treatment-AQ_Control	-0.013147022	-0.106649368	0.0803553229	0.9999984
CO_Control-AQ_Treatment	-0.182420129	-0.275922474	-0.0889177836	0.0000002
CO_Treatment-AQ_Treatment	-0.200237276	-0.293739621	-0.1067349305	0.0000000
CY_Control-AQ_Treatment	-0.294646696	-0.388149042	-0.2011443510	0.0000000
CY_Treatment-AQ_Treatment	-0.233365573	-0.326867918	-0.1398632274	0.0000000
IB_Control-AQ_Treatment	-0.348313299	-0.441815644	-0.2548109536	0.0000000
IB_Treatment-AQ_Treatment	-0.418931240	-0.512433586	-0.3254288951	0.0000000
IR_Control-AQ_Treatment	-0.145654430	-0.239156775	-0.0521520847	0.0000645
IR_Treatment-AQ_Treatment	-0.229824876	-0.323327221	-0.1363225305	0.0000000
ST_Control-AQ_Treatment	-0.098801457	-0.192303802	-0.0052991114	0.0288267
ST_Treatment-AQ_Treatment	-0.049595368	-0.143097713	0.0439069777	0.8261911
CO_Treatment-CO_Control	-0.017817147	-0.111319492	0.0756851985	0.9999635
CY_Control-CO_Control	-0.112226567	-0.205728913	-0.0187242220	0.0061545
CY_Treatment-CO_Control	-0.050945444	-0.144447789	0.0425569015	0.8000257
IB_Control-CO_Control	-0.165893170	-0.259395515	-0.0723908246	0.0000029
IB_Treatment-CO_Control	-0.236511111	-0.330013457	-0.1430087661	0.0000000
IR_Control-CO_Control	0.036765699	-0.056736646	0.1302680443	0.9748654
IR_Treatment-CO_Control	-0.047404747	-0.140907092	0.0460975984	0.8645319
ST_Control-CO_Control	0.083618672	-0.009883673	0.1771210176	0.1259268
ST_Treatment-CO_Control	0.132824761	0.039322416	0.2263271067	0.0004103
CY_Control-CO_Treatment	-0.094409420	-0.187911766	-0.0009070752	0.0456199
CY_Treatment-CO_Treatment	-0.033128297	-0.126630642	0.0603740484	0.9888108
IB_Control-CO_Treatment	-0.148076023	-0.241578368	-0.0545736777	0.0000450
IB_Treatment-CO_Treatment	-0.218693965	-0.312196310	-0.1251916192	0.0000000
IR_Control-CO_Treatment	0.054582846	-0.038919500	0.1480851911	0.7212640
IR_Treatment-CO_Treatment	-0.029587600	-0.123089945	0.0639147453	0.9956340
ST_Control-CO_Treatment	0.101435819	0.007933474	0.1949381644	0.0216375

ST_Treatment-CO_Treatment	0.150641908	0.057139563	0.2441442536	0.0000306
CY_Treatment-CY_Control	0.061281124	-0.032221222	0.1547834689	0.5568318
IB_Control-CY_Control	-0.053666603	-0.147168948	0.0398357428	0.7421079
IB_Treatment-CY_Control	-0.124284544	-0.217786889	-0.0307821987	0.0013170
IR_Control-CY_Control	0.148992266	0.055489921	0.2424946116	0.0000392
IR_Treatment-CY_Control	0.064821820	-0.028680525	0.1583241658	0.4683598
ST_Control-CY_Control	0.195845240	0.102342894	0.2893475849	0.0000000
ST_Treatment-CY_Control	0.245051329	0.151548983	0.3385536741	0.0000000
IB_Control-CY_Treatment	-0.114947726	-0.208450071	-0.0214453808	0.0043973
IB_Treatment-CY_Treatment	-0.185565668	-0.279068013	-0.0920633223	0.0000001
IR_Control-CY_Treatment	0.087711143	-0.005791203	0.1812134881	0.0874065
IR_Treatment-CY_Treatment	0.003540697	-0.089961648	0.0970430423	1.0000000
ST_Control-CY_Treatment	0.134564116	0.041061771	0.2280664614	0.0003213
ST_Treatment-CY_Treatment	0.183770205	0.090267860	0.2772725505	0.0000002
IB_Treatment-IB_Control	-0.070617941	-0.164120287	0.0228844038	0.3345757
IR_Control-IB_Control	0.202658869	0.109156524	0.2961612142	0.0000000
IR_Treatment-IB_Control	0.118488423	0.024986078	0.2119907684	0.0028094
ST_Control-IB_Control	0.249511842	0.156009497	0.3430141875	0.0000000
ST_Treatment-IB_Control	0.298717931	0.205215586	0.3922202766	0.0000000
IR_Control-IB_Treatment	0.273276810	0.179774465	0.3667791557	0.0000000
IR_Treatment-IB_Treatment	0.189106365	0.095604019	0.2826087099	0.0000001
ST_Control-IB_Treatment	0.320129784	0.226627438	0.4136321290	0.0000000
ST_Treatment-IB_Treatment	0.369335873	0.275833527	0.4628382181	0.0000000
IR_Treatment-IR_Control	-0.084170446	-0.177672791	0.0093318995	0.1200576
ST_Control-IR_Control	0.046852973	-0.046649372	0.1403553186	0.8733440
ST_Treatment-IR_Control	0.096059062	0.002556717	0.1895614078	0.0385066
ST_Control-IR_Treatment	0.131023419	0.037521074	0.2245257644	0.0005271
ST_Treatment-IR_Treatment	0.180229508	0.086727163	0.2737318536	0.0000003
ST_Treatment-ST_Control	0.049206089	-0.044296256	0.1427084345	0.8333861

Table S5. Statistical output of PERMANOVA (adonis2, vegan package) testing the effect of sampling time on the microbiome composition for each host species within treatment groups (10 000 permutations).

```
adonis2(formula = d ~ SamplingTimepoint, data = df, permutations = perm,
method = "bray")
```

	Df	SumOfSqs	F	Pr(>F)
SamplingTimepoint	2	0.197	0.218	0.9988
Residual	105	47.372		

Table S6. Statistical output of PERMANOVA (adonis2, vegan package) testing the effect of host genotype on the microbiome composition within each host species (10 000 permutations).

```
adonis2(formula = d ~ Genotype, data = df, permutations = perm, method
= "bray")
```

	Df	SumOfSqs	F	Pr(>F)	
Genotype	35	42.356	16.714	9.999e-05	***
Residual	72	5.213			

References

Lichtenthaler, H. . 1987. "Chlorophylls and Carotenoids - Pigments of photosynthetic biomembranes." *Methods Enzymol* 148:350-382.

Ritchie, R. 2008. "Universal chlorophyll equations for estimating chlorophylls a, b, c, and d and total chlorophylls in natural assemblages of photosynthetic organisms using acetone, methanol, or ethanol solvents." *Photosynthetica* 46:115-126.
